# GBF/Gea mutant with a single substitution sustains fungal growth in the absence of BIG/Sec7

**DOI:** 10.1016/j.febslet.2014.11.014

**Published:** 2014-12-20

**Authors:** Herbert N. Arst, Miguel Hernandez-Gonzalez, Miguel A. Peñalva, Areti Pantazopoulou

**Affiliations:** a Section of Microbiology, Department of Medicine, Imperial College London, London SW7 2AZ, United Kingdom; b Centro de Investigaciones Biológicas, CSIC, Madrid 28040, Spain

**Keywords:** Arf GTPases, ADP-ribosylation-factor GTPases, BFA, brefeldin A, BIG, brefeldin A-inhibited guanine nucleotide-exchange, GBF, Golgi-specific brefeldin A resistance factor, GEF, Guanine nucleotide exchange factor, PH^OSBP^, pleckstrin homology domain of the oxysterol binding protein, Sec7d, Sec7 domain, Golgi Arf1-GEFs, GBF/Gea-subfamily, BIG/Sec7-subfamily, Fungal secretion

## Abstract

Golgi Arf1-guanine nucleotide exchange factors (GEFs) belong to two subfamilies: GBF/Gea and BIG/Sec7. Both are conserved across eukaryotes, but the physiological role of each is not well understood. *Aspergillus nidulans* has a single member of the early Golgi GBF/Gea-subfamily, *geaA*, and the late Golgi BIG/Sec7-subfamily, *hypB*. Both *geaA* and *hypB* are essential. *hypB*5 conditionally blocks secretion. We sought extragenic *hypB*5 suppressors and obtained *geaA*1. *geaA*1 results in Tyr1022Cys within a conserved GBF/Gea-specific S(Y/W/F)(L/I) motif in GeaA. This mutation alters GeaA localization. Remarkably, *geaA*1 suppresses *hypB*Δ, indicating that a single mutant Golgi Arf1-GEF suffices for growth.

## Highlights

• *A. nidulans* has a GBF/Gea and a BIG/Sec7 subfamily Golgi Arf1-GEFs, both essential.
• The late Golgi Arf1-GEF mutant *hypB*5 conditionally blocks secretion.
• Residue substitution in the early Golgi Arf1-GEF GeaA suppresses *hypB*5 *and hypB*Δ.
• The mutation alters a GBF/Gea amino acid motif and shifts GeaA localization.
• GeaA1 alone satisfies the eukaryotic requirement for two Golgi Arf1 GEFs.

## 1 Introduction

ADP-ribosylation-factor (Arf) GTPases regulate membrane traffic by organizing vesicle budding. Their activation depends on the GEF (Guanine nucleotide Exchange Factor)-catalyzed exchange of GTP for GDP, that leads to a conformational switch stabilizing membrane insertion of their N-terminal myristoylated amphipathic helix and facilitating recruitment of specialized effectors [1], [2].

Arf1, an essential Golgi regulator, is activated by the GBF/Gea and the BIG/Sec7 GEF subfamilies [1]. These two subfamilies comprise related proteins sharing the highly conserved catalytic Sec7 domain (Sec7d) and five conserved regions denoted DCB, HUS, HDS1, -2 and -3 domains [3], [4], [5]. GBF/Gea and BIG/Sec7 are both considered essential for Golgi function in *Saccharomyces cerevisiae*, *Drosophila melanogaster* and mammalian cells [6], [7], [8], [9], [10], [11], [12], [13], [14]. Moreover, they are the only Arf-GEF subfamilies common to all eukaryotes [3]. Thus, although both GBF/Gea and BIG/Sec7 activate Arf1 at the Golgi, they have some non-overlapping essential functions.

Apart from triggering Arf1-mediated effector recruitment, Arf1-GEFs each engage specific protein interactors, resulting in variable outcomes from the activation of a single Arf. This possibly underlies non-overlapping Arf1-GEF functions. For example, Gea1p/GBF1 interacts with Sec21p/γ-COP [15].

Arf GEFs are peripheral membrane proteins: their membrane recruitment is tightly regulated to ensure precise spatiotemporal responses [16], [17], [18], [19]. However, the mechanistic bases of this regulation are incompletely understood [reviewed in [20]]. It is widely accepted that the BIG/Sec7 subfamily members act at the late/*trans*-Golgi, while the GBF/Gea subfamily members act at the early/*cis*-Golgi [1]. An HDS1-mediated interaction of *S. cerevisiae* Sec7p with membrane-bound Arf1-GTP contributes to Sec7p recruitment to, and activation at late Golgi compartments via a positive feedback loop [21]. The Arf-like protein Arl1 is necessary for recruitment of the Sec7-orthologues BIG1/2 at the mammalian *trans*-Golgi [22]. Mammalian GBF1 is a Rab1 effector [23]. Its *cis*-Golgi recruitment occurs in response to an increase in membrane-associated Arf-GDP [24]. The HDS1 domain of its *S. cerevisiae* homologue Gea1p was implicated in lipid droplet binding and Golgi recruitment [25] and in interaction with the early Golgi resident Gmh1p [26]. Coincidence detection might additionally link the above observations and determine the Arf1-GEFs localizations at the Golgi.

How different sets of interactors and/or localization determinants result in essentially distinct functions of the two Golgi Arf1-GEF subfamilies and how these functions serve cargo passage through the Golgi are unanswered questions.

## 2 Materials and methods

### 2.1 Deletion of hypB

**Fig. 1:**
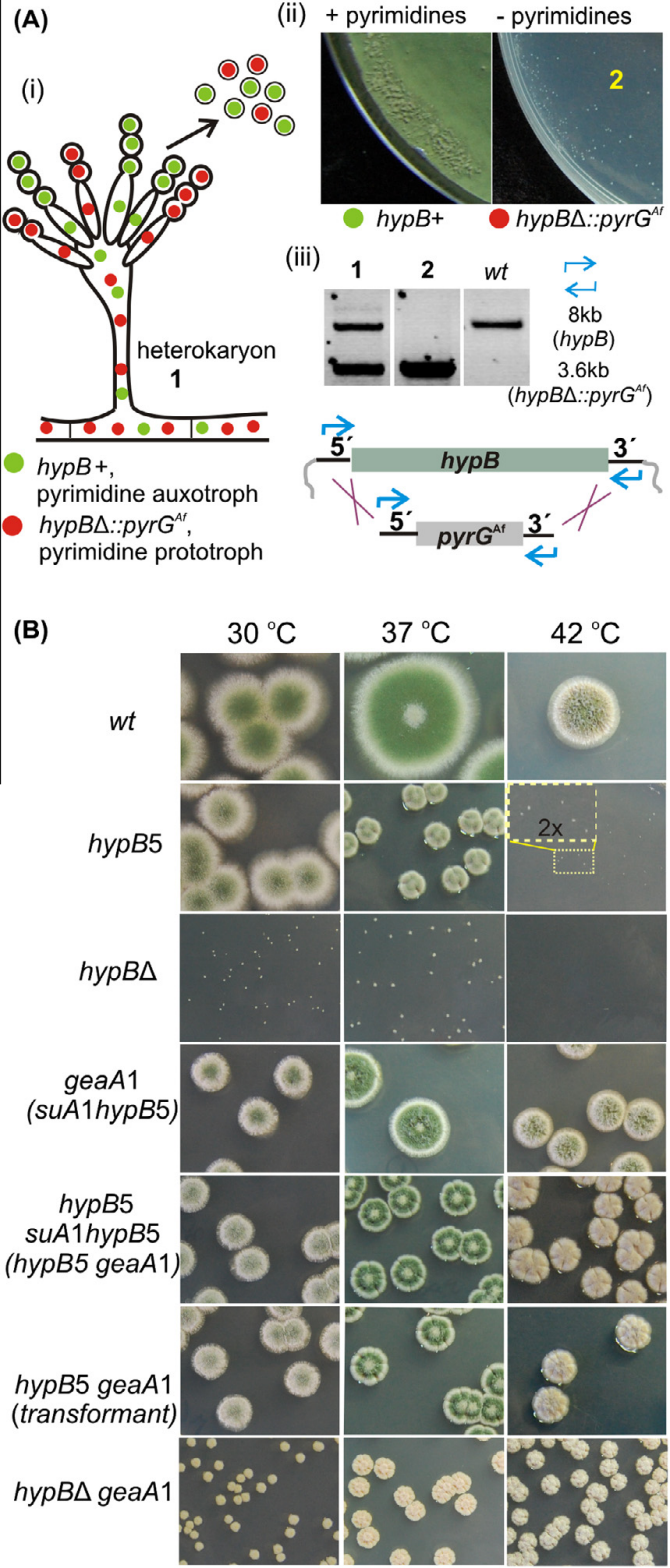
(A) Deletion of the *hypB* gene encoding the only *A. nidulans* late-Golgi Arf1-GEF is virtually lethal. *hypB*Δ transformants, selected as pyrimidine prototrophs, were heterokaryotic (i), i.e., they carried both transformed (*hypB*Δ:: *pyrG^Af^*) and wild type (*hypB *+ *pyrG89*) nuclei. Spores produced by these heterokaryons are uninucleate. On medium without pyrimidines, untransformed nuclei-containing spores cannot grow due to pyrimidine auxotrophy, which permits testing for growth of the prototroph spores that contain the deletion mutation. *hypB*Δ spores form aconidial microcolonies (ii). PCR genotyping with DNA from the multinuclear primary transformant mycelium (1) yielded amplification bands corresponding to both the wild type *hypB* and the *hypB*Δ::*pyrG^Af^* deletion cassette (iii), confirming that this was heterokaryotic. In contrast, only the band corresponding to the *hypB*Δ::*pyrG^Af^* construct was amplified from DNA from the micro-colonies on the selective medium (2), indicating that these are homokaryotic *hypB*Δ::*pyrG^Af^* colonies, which showed that HypB is virtually essential for *A. nidulans*. (B) GBF/Gea-subfamily mutant GeaA1 bypasses the requirement for the BIG/Sec7-subfamily HypB. Growth at the specified temperatures from spore dilutions of strains: *wt* (wild type, MAD2), *hypB*5 (MAD3574), *hypB*Δ (*hypB*Δ::*pyrG^Af^ pyrG*89; *nkuA*Δ::*bar pyroA*4), *geaA*1 (MAD4062), *hypB*5 *suA*1*hypB*5 (MAD4041-obtained by UV mutagenesis), *hypB*5 *geaA*1 transformant (MAD4836-obtained by transformation of a *hypB*5 strain with a DNA fragment carrying the *geaA*1 mutation) and *hypB*Δ *geaA*1 (MAD5130). All photos are at the same magnification (except the one indicated as 2×, this is a 2 times magnification of the yellow rectangular region).

*hypB* is AN6709 (http://www.aspgd.org/). We constructed by fusion PCR (primers 14–19, Supplementary Table II) [27], [28], a cassette (Fig. 1) for substituting the *hypB* ORF with *Aspergillus fumigatus pyrG* in wt (MAD1739-List of strains in Supplementary Table I) and *geaA1* backgrounds (MAD5107). The cassette was cloned in pGEM (plasmid p2001) and checked by sequencing. A p2001 linearized (NcoI/NsiI) fragment was used in transformations for gene replacement. We assessed the lethality of *hypB* deletion using the heterokaryon rescue [29]. Diploids, heterokaryons and homokaryotic mini-colonies (*hypB*Δ) or non-sporulating colonies (*hypB*Δ *geaA1*) of transformants were genotyped by PCR (primers 14 and 19) and/or by Southern blots.

### 2.2 Ultraviolet light (UV) induced mutagenesis and molecular characterization of suppressors

40 UV-induced *hypB*5 suppressors were selected in MAD3574 for growth at 42 °C. In all but one case, reversion occurred within *hypB* (Table 1). Meiotic crosses verified that this suppressor mutation (*suA*1*hypB*5) is extragenic. Haploidization analysis [30] localized the suppressor mutation to chromosome VIII. We meiotically mapped *suA*1*hypB*5 to a position between *nudA* and *nirA* (In the process of mapping *suA*1*hypB*5, we identified the mutational lesion of *sE*15 as a frameshift in *trxA*, see Supplementary Materials and Methods). AN0112, encoding GeaA, was identified as the most likely candidate in this interval and was sequenced (primers 20–27).

**Table 1:** Revertants of *hypB*5.

Strain		HypB aminoacid sequence^a^
*hypB*5		A881P
Revertants		
Revertant type	Times obtained	
True revertant	1	A881
First site S	19	A881S
First site L	12	A881L
First site T	2	A881T
First site T and second site	1	D880G A881T
Second site	2	T863I A881P
Second site	1	A881P N956L
Second site	1	ΔG854ΔE855 A881P
Extragenic (*hypB*5 *geaA*1)^b^	1	A881P
	Total = 40	

a Changes in comparison with the wild type strain MAD2. Only the nucleotide sequence coding for F825 to D961 of HypB was determined (amplified with primers 1 and 2), except in the case of the “extragenic” suppressor, where the whole *hypB* was sequenced (primers 1 to 13 and 20) and found identical to *hypB*5 parent.

b MAD4041.

### 2.3 Reconstruction of the *hypB*5 geaA1 strain by transformation

To confirm that the suppression phenotype in *suA*1*hypB*5 is indeed due to the mutation identified in *geaA* (*geaA*1), we PCR-amplified (from MAD4041 gDNA using primers 22 and 23) an ∼1.5 kb region of *geaA*1 carrying the A3065G substitution (approximately in the middle of the fragment) and used this molecule to transform *hypB*5 strain MAD3574. We directly selected transformants for growth at 42 °C, the restrictive temperature for *hypB*5, and verified by sequencing that all transformants contained *geaA*1. This procedure yielded MAD4836.

### 2.4 In silico analyses

Sequences used for in silico analyses that detected the GBF/Gea-specific motif were identified by Blast using as queries the *S. cerevisiae* Gea1p (GenBank CAA89558.1)/Sec7p (NCBI NP_010454.3), *Aspergillus nidulans* GeaA (AN0112)/HypB (AN6709) or *Homo sapiens* GBF1 (GenBank AAI17683.1)/BIG1 (NCBI NP_006412.2). Alignments used T-Coffee (http://tcoffee.crg.cat/apps/tcoffee/do:regular), while their visualization and editing were performed with GeneDoc.

### 2.5 GFP-tagging and microscopy

Using PCR (primers 30 through 39), we fused part of the *geaA* ORF (starting downstream the nucleotide that is mutated in *geaA*1) to *gfp* in frame, the 3′UTR of *geaA* and *pyrG* of *A. fumigatus* (Fig. 3). This fragment was cloned in pGEM generating p2198 and was sequenced to verify the absence of mutations. p2198 was linearized (BglII/SpeI) to transform MAD1739 for obtaining *geaA*::*gfp* or MAD5107 for *geaA*1::*gfp*. Transformants were analyzed by Southern blot to verify *in locus* integration.

*In vivo* microscopy used an inverted Leica DMI6000B microscope [optics/procedures as previously described in [31]]. Deconvolution was done with Huygens Professional (www.svi.nl). Further image processing was with MetaMorph (Molecular Devices). FM4-64 staining was as previously described [32].

Co-localization was studied with Li’s Intensity Correlation Analysis [33] and the Pearson’s correlation coefficient using the JACoP plugin [34] of ImageJ (http://imagej.nih.gov/ij/). Maximal intensity projections of 3-plane deconvolved *z*-stacks (total width = 600 nm) were used. Regions for co-localization assays were selected as described in [31]. Due to the difference in intensities of the channels, image stacks in each channel were acquired consecutively using different exposure times. Co-localization was nevertheless possible because low motility of Golgi cisternae makes the time required for shifting between the two channels irrelevant. Statistical analyses of correlation coefficients was based on [35] and used the GraphPad Prism 6 (www.graphpad.com).

## 3 Results and discussion

The *A. nidulans* genome codes for a single member of each of the two Arf-GEF subfamilies. AN0112 encodes the GBF/Gea homologue, GeaA. HypB (encoded by AN6709, www.aspgd.org) is the BIG/Sec7 homologue [36], co-localizing with the Arf1- and PtIns4P-binding, late-Golgi marker mRFP-PH^OSBP^ [37], [38]. HypB/mRFP-PH^OSBP^-containing cisternae are resolvable from Sed5-containing early Golgi cisternae in the unstacked *A. nidulans* Golgi [39], [40]. Late Golgi cisternae are transient and mature to RabE^Rab11^ post-Golgi carriers [41].

**Supplementary Fig. S1:**
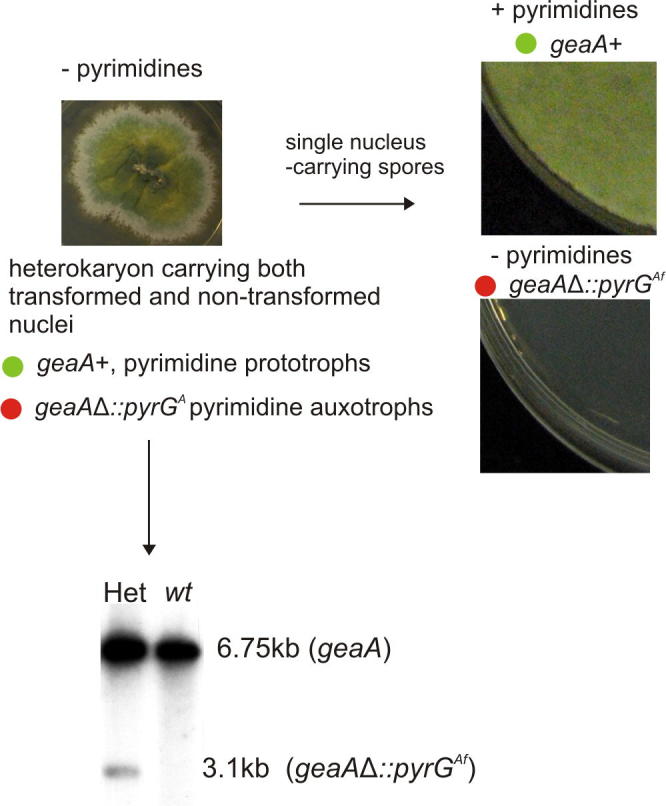
Deletion of the geaA gene encoding the only *A. nidulans* early-Golgi Arf1-GEF is lethal. MAD1739 recipient strain was transformed with a linear DNA fragment containing the *A. fumigatus* pyrG flanked by the *geaA* 5′ and 3′UTRs, resulting in locus integration and replacement of the *geaA* coding region by *pyrG*. Primary transformants, selected as pyrimidine prototrophs, were heterokaryotic, carrying both transformed (*geaA*Δ::*pyrG^Af^*) and wild type (geaA+, pyrG89) nuclei. The presence of both nuclei in the primary transformants was confirmed by Southern blot, showing both a band of the size corresponding to the wild type *geaA*, as well as the higher mobility band corresponding to *geaA*Δ::*pyrG^Af^*. Spores produced by heterokaryonsare uninucleate. On medium without pyrimidines no growth of the prototroph *geaA*Δ::*pyrG^Af^* spores was detected, indicating that GeaA is essential.

Fungal life is crucially dependent on exocytosis, required to maintain the cell wall. The *hypB*Δ null mutation severely impairs hyphal growth [36]. Using heterokaryon rescue [29] (Fig. 1A), we found that *hypB*Δ spores give rise to aconidial microcolonies on solid medium at 30 and 37 °C, but they do not grow at 42 °C (Fig. 1A and B). We conclude that *hypB*Δ is virtually lethal. Spores carrying the *geaA*Δ null mutation are also inviable (Supplementary Fig. 1) and, thus, both HypB and GeaA are essential for fungal growth.

**Fig. 2:**
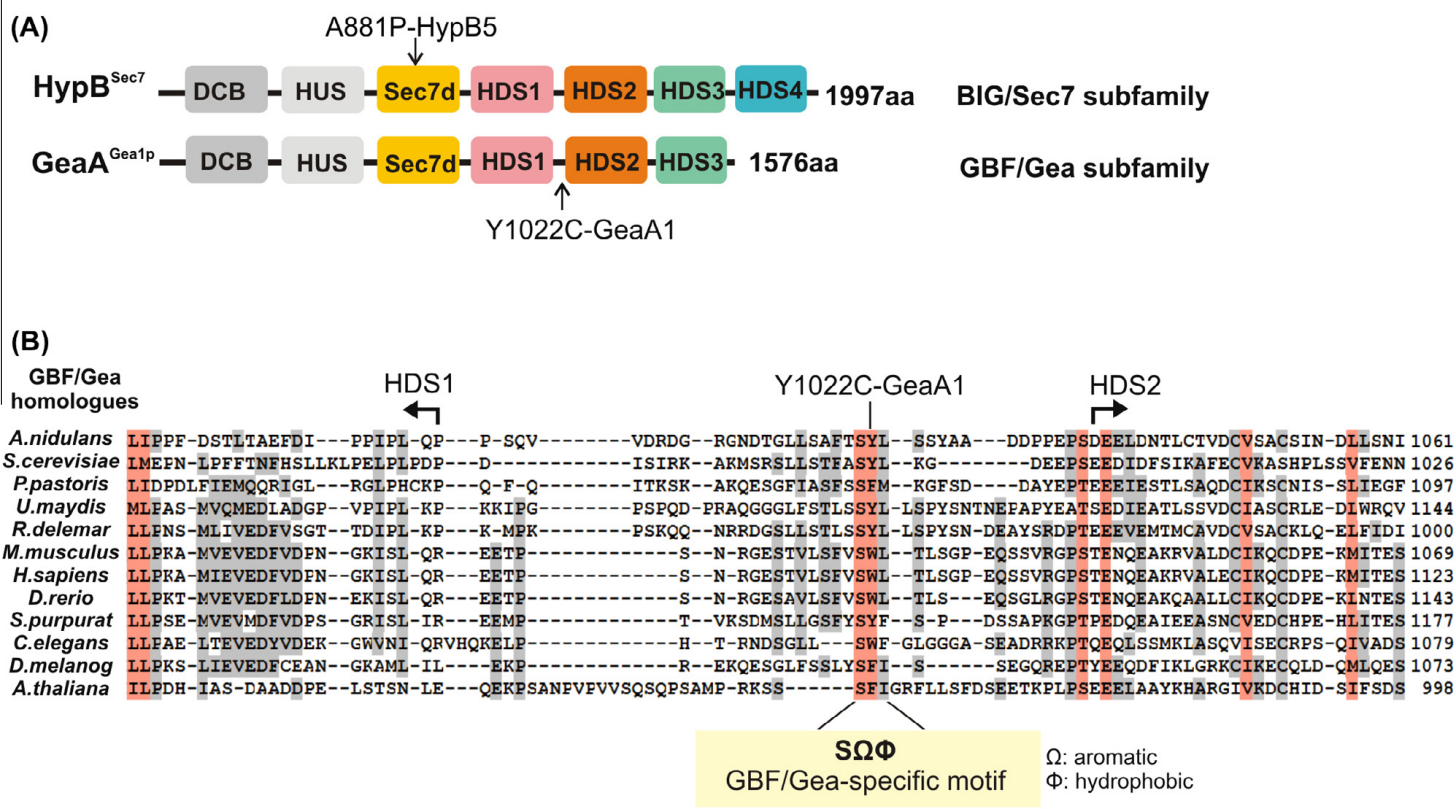
GeaA1-Y1022C substitution lies in a region between HDS1 and HDS2 and alters a conserved GBF/Gea-specific motif. (A) Upper panel: scheme of HypB sequence homology domains [named DCB, HUS, Sec7d, HDS1-HDS4 [3], [4], identified by T-coffee alignment with yeast and mammalian homologue sequences]. HypB5 (A881P) affects a conserved amino acid residue within the catalytic Sec7d. Lower panel: scheme of GeaA sequence homology domains. GeaA-Y1022C lies between HDS1 and HDS2. (B) Summary of multiple alignments of GBF/Gea homologues from Opisthokonta and *Arabidopsis thaliana* in the region between HDS1 and HDS2, where GeaA1-Y1022C lies (sequences displayed are from: *A. nidulans* XP_657716, *Saccharomyces cerevisiae* CAA89558, *Pichia pastoris* XP_002490563, *Ustilago maydis* XP_757309, *Rhizopus delemar* EIE78027, *Mus musculus* BAD32197, *Homo sapiens* NP_004184, *Danio rerio* XP_694714, *Strongylocentrotus purpuratus* XP_003728128, *Caenorhabditis elegans* NP_001255140, *Drosophila melanogaster* NP_610761, *A. thaliana* NP_198766). We looked for robust alignment of HDS1 and 2. Then we inspected and manually adjusted the less conserved region between HDS1 and HDS2. 11 sequences of Aspergilli GBF/Gea invariably contain serine, tyrosine (Y1022 in GeaA), leucine (SYL) in the region between HDS1 and 2. In 27 further fungal sequences, including basidiomycetes, zygomycetes, ascomycetes, the motif is SY (L/I/V), with few exceptions (*P. pastoris*: SFM, *Candida albicans*: SFL). >30 sequences of vertebrates (including mammals, zebrafish, xenopus) contain a SFVSWL sequence, corresponding to a duplication of the motif. Echinodermata have SYF. In ecdysozoa, Drosophila has SFI, *Ceratitis capitata* and *Bombyx mori* have SY L/I, *C. elegans* has SWF. We conclude that a serine-aromatic-hydrophobic aminoacid motif, SΩΦ (yellow box), is conserved in GBF/Gea proteins in Opisthokonta. The *A. thaliana* GBF-subfamily member GLN1, functioning at the Golgi [53], possesses SFI between HDS1 and HDS2. On the contrary, among 30 BIG/Sec7 subfamily fungal members tested, only *S. cerevisiae* Sec7p and the *Ashbya gossypii* and *Candida albicans* Sec7 homologues contain SFF, a GBF/Gea-motif related sequence, in the region between HDS1 and HDS2 (no SFF motif variant is found in any GBF/Gea member), while no motif was found in BIG members of vertebrates/ecdysozoa.

*hypB*5 is a *ts* mutation [42], [43], resulting in Ala881Pro in the catalytic Sec7d [36] (Fig. 2A). *hypB*5 strains grow well at 30 °C but form aconidial microcolonies at 42 °C (Fig. 1B). Ala881 lying in a conserved region of the Sec7d α-helix F contributes to a hydrophobic network situated across from the catalytic hydrophobic groove that contacts Arf1 [44]. By interfering (putatively) with the folding of α-helix F, Ala881Pro might decrease HypB stability at elevated temperatures. Shifting *hypB*5 cells from 28 °C to 37 °C results in cessation of apical extension and mislocalization of exocytic-carriers from an apical crescent to intracellular structures [31], [41], [45], showing that exocytosis is prevented.

To determine whether the essential late Golgi function of HypB for secretion can be bypassed, we sought suppressors of *hypB*5 at 42 °C. Out of 40 strains able to grow at 42 °C, one extragenic mutation, *suA*1*hypB*5, was obtained (Table 1)*.* Suppression of *hypB*5 is partial (Fig. 1B) and *suA*1*hypB*5 itself results in constricted growth (Fig. 1B) and is dominant in diploids. Genetic analyses localized it between AN0118 (*nudA*) and AN0098 (*nirA*). The *nudA* to *nirA* 75 kb-interval contains 24 autocalled genes including AN0112 encoding the early Golgi Arf1-GEF homologue, GeaA. Sequencing of AN0112 in *suA*1*hypB*5 revealed the presence of a missense A3065G mutation (*geaA1*) resulting in Tyr1022Cys (GeaA1). Reconstruction by transformation of the *geaA*1 mutation in a *hypB*5 strain showed that *geaA*1 suppresses *hypB*5^ts^ (i.e., *geaA*1 is indeed *suA*1*hypB*5) (Fig. 1B).

To determine whether *geaA*1 also suppresses *hypB*Δ, we deleted *hypB* in a *geaA*1 strain and, in contrast to the heterokaryotic transformants obtained when *hypB* was deleted in the wild type background (Fig. 1A), we recovered homokaryotic *hypB*Δ *geaA*1 double mutants, showing that HypB is dispensable in the *geaA*1 genetic background (Fig. 1B).

GeaA-Tyr1022 lies between the HDS1 and HDS2 domains (Fig. 2A and B) [3], [4]. Using fungal GBF/Gea and BIG/Sec7 homologues in multiple alignments, we found that Tyr1022 lies in a previously unidentified Gea-specific motif Ser-Ω-Φ, where Ω indicates any aromatic and Φ any hydrophobic residue [fungal consensus: SYL]. The Ser-Ω-Φ motif is conserved in GBF/Gea of both vertebrates and ecdysozoa (Fig. 2B).

The finding that the substitution alters a conserved GBF/Gea-specific motif suggests that it is the (partial or total) loss of one GEA-specific function in GeaA1 (GeaA^Y1022C^) that makes SEC7-specific functions dispensable. Members of the GBF/Gea subfamily, although predominating at the early Golgi [4], have been recently reported to localize also at the *trans*-Golgi and TGN [46], [47]. An attractive hypothesis was that Tyr1022Cys shifts GeaA1 localization towards the late Golgi, perhaps by reducing its affinity for an early Golgi receptor, and that this suffices to bypass HypB partially.

**Fig. 3:**
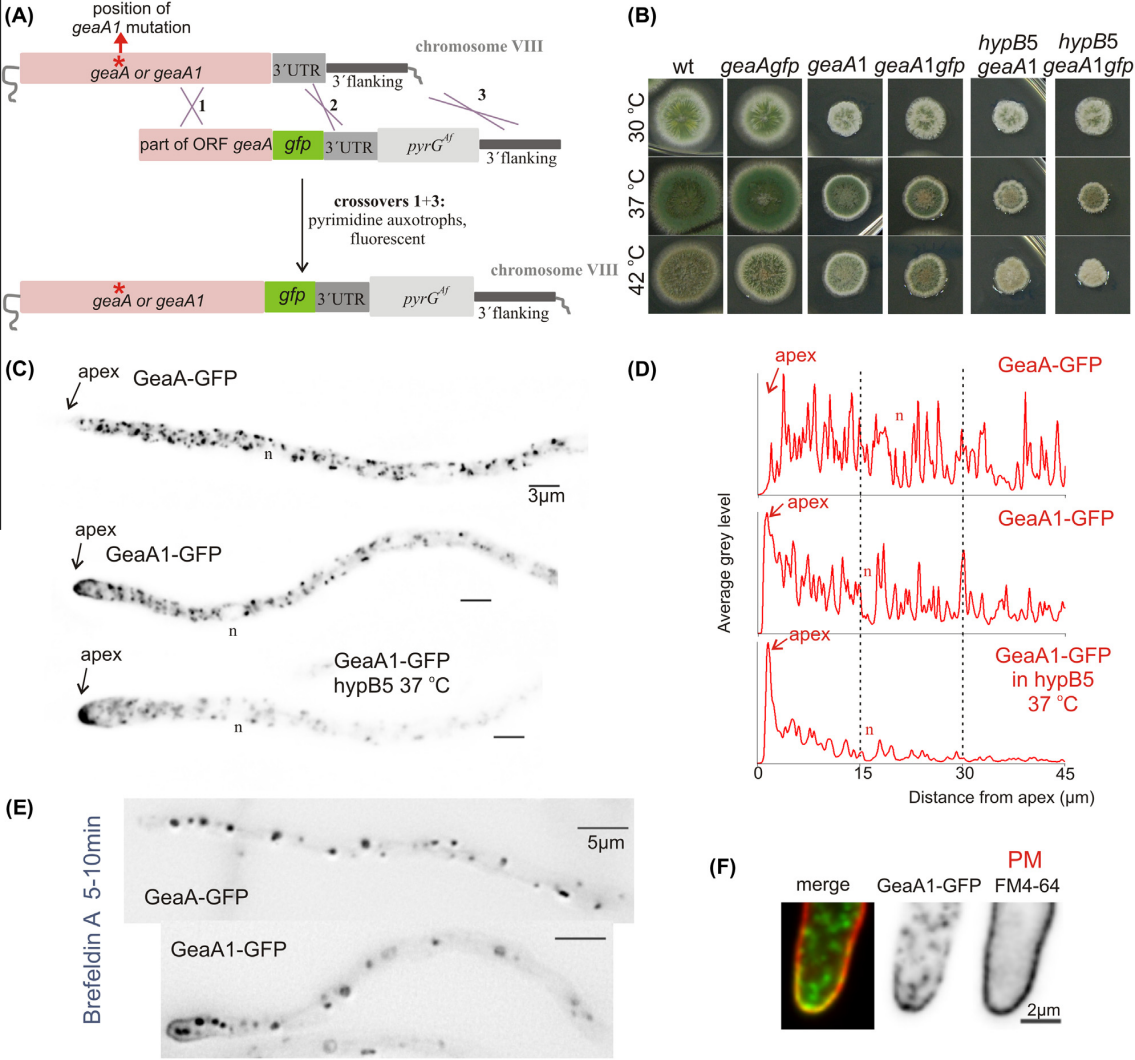
Intracellular distribution of GeaA-GFP and GeaA1-GFP. (A) C-terminal tagging of *geaA* or *geaA*1 with *gfp* was achieved by *in locus* integration by transformation of a linear cassette containing part of the *geaA* open reading frame fused to *gfp*, the *geaA* 3′UTR and the *A. fumigatus pyrG* as a selection marker, resulting in strains carrying a single *gfp*-tagged copy of the *geaA* or *geaA*1 allele. (B) Growth test showing that GFP tagged GeaA and GeaA1 are functional. Note that GeaA1-GFP suppresses *hypB*5 thermosensitivity, like the untagged allele. (C) Maximal intensity projections of deconvolved *z*-stacks. GeaA-GFP localizes at Golgi cisternae. GeaA1-GFP, although still localizing at Golgi cisternae, also labels an apical crescent and an apex-associated accumulation. The apical localization is maintained in the *hypB*5 background after shift to 37 °C for 40 min (*n*: nucleus, bar: 3 μm). (D) Fluorescence intensity profiles across the hyphae in (C) (*n*: position of the nucleus). Note the shift towards the apex displayed by GeaA1-GFP, compared to GeaA-GFP. (E) Hyphae treated with 200 μg/ml BFA for the indicated time, a treatment that provokes the collapse of the Golgi into large aggregates. Note that in GeaA1-GFP some labeling of the apical crescent is still visible in these conditions. (F) Staining of the plasma membrane with FM4-64 in a GeaA1-GFP-expressing strain shows that GeaA1 in the apical crescent is at or very closely associated with the plasma membrane.

**Fig. 4:**
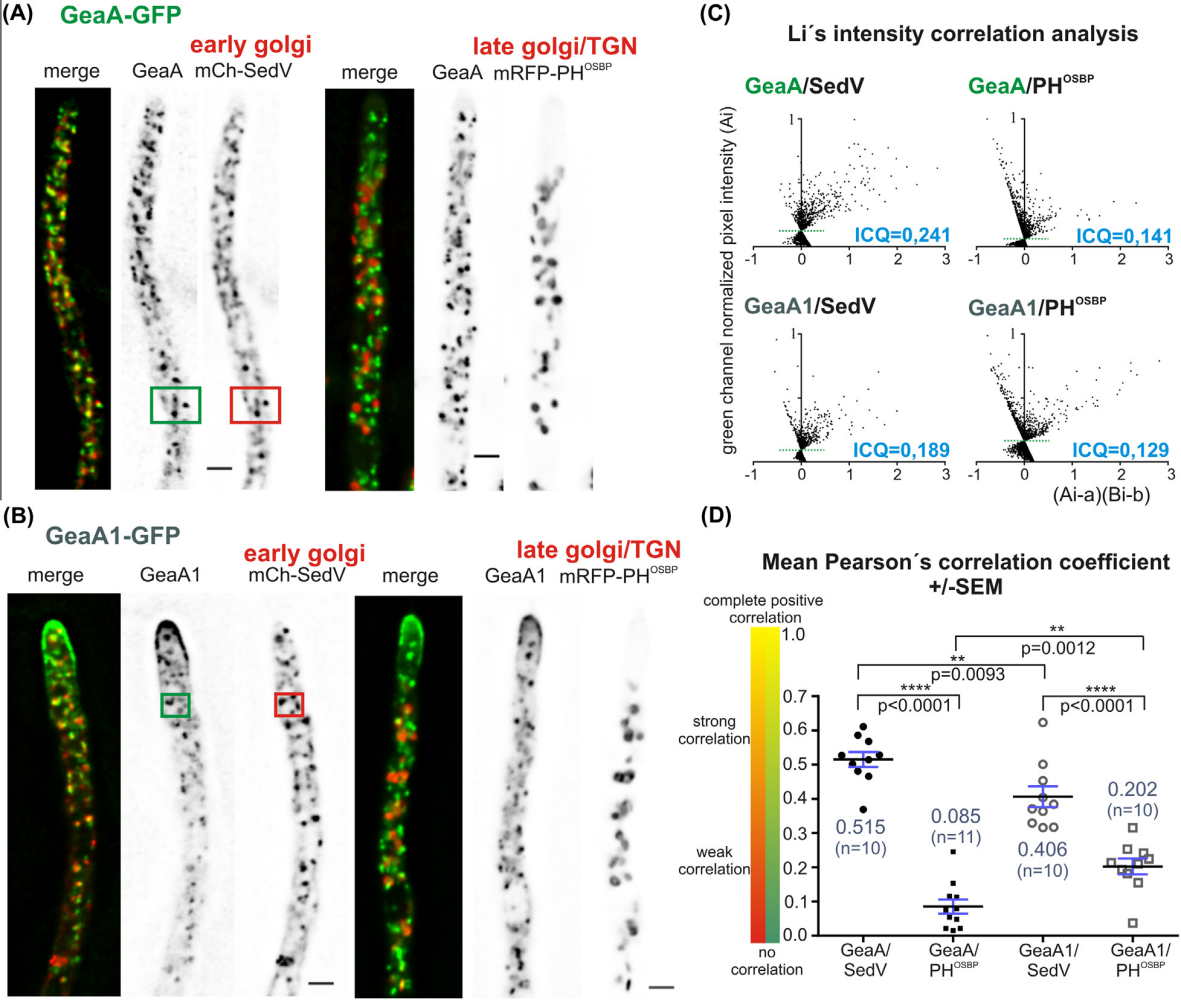
Intra-Golgi distribution of GeaA-GFP and GeaA1-GFP. Maximal intensity projections of deconvolved *z*-stacks, using strains co-expressing GeaA-GFP and the early Golgi marker mCherry-SedV (left panel) or the late Golgi marker mRFP-PH^OSBP^ (right). Note the high degree of overlap (yellow color in the “merge” image) between GeaA-GFP and mCherry-SedV, contrasting the low overlap of GeaA-GFP and mRFP-PH^OSBP^. Coincidence in shape of fluorescent structures indicated true co-localization (examples included in the green and red boxes). Bar = 2 μm. (B) As in (A), but with strains expressing mutant GeaA1-GFP. (C) Li’s green channel intensity correlation analysis of the indicated pair of markers. The analysis was done for a region of the cells displayed in (A and B). The *y*-value reflects the intensity distribution in the GFP channel. The *x*-value depends on co-variance of the two channels. The cloud of points towards the right of the *y* axis (positive values) reflects positive correlation of the two channels (co-localization). The horizontal dotted green line intersects the *y* axis at the mean intensity value. Note that, in the GeaA/SedV graph, the majority of the “above the average intensity” pixels form a cloud on the right of the *y* axis, indicating localization of GeaA to the early Golgi. The opposite is true for GeaA/PH^OSBP^, where high intensity pixels have negative *x*-values, indicating inverted correlation (ICQ: the intensity correlation quotient. ICQ approaches 0.5 in complete co-localization or 0.0 in random staining) (Ai: *gfp* pixel intensity, *a*: *gfp* average pixel intensity, Bi: rfp pixel intensity, *b*: rfp average pixel intensity). (D) Graph showing the dispersion and the mean value of the Pearson’s correlation coefficient (PCC) calculated from co-localization analysis in (*n*) examples for each pair of markers. Blue bars indicate the standard error of the mean. The difference between the mean PCC of the pair GeaA/SedV versus the mean PCC of GeaA/PH^OSBP^ is large and very significant, showing that GeaA-GFP mainly localizes at the early Golgi. The difference in PCC between GeaA1/SedV versus GeaA1/PH^OSBP^ is also large, suggesting that the mutant GeaA1-GFP also predominates at the early Golgi. However, a drop in the mean PCC of GeaA1/SedV compared to GeaA/SedV suggests that localization of mutant GeaA1 at the early Golgi is reduced. This correlates with GeaA1 localization shift to the apical crescent in (B). A small increase in PCC of GeaA1/PH^OSBP^ versus GeaA/PH^OSBP^ is also observed, although the correlation between the two markers is still weak in the mutant GeaA1.

Therefore, we investigated GeaA localization, replacing the endogenous *geaA* coding region by the *geaA*::*gfp* fusion allele (Fig. 3A). GeaA-GFP is functional, as shown by almost wild type growth of a strain carrying solely the *geaA*::*gfp* allele (Fig. 3B). GeaA-GFP labels punctate cytosolic structures resembling in shape and polarization towards the tip the *A. nidulans* Golgi cisternae (Fig. 3C and D) [38]. In growing hyphae, GeaA-GFP structures are found in the proximity of the apex, like early Golgi cisternae (late Golgi are excluded from the most proximal sub-apical area) [38], [39]. GeaA-GFP is also partly cytosolic, as suggested by the fact that nuclei are visible as “empty” hollows against the fluorescent cytosolic background (Fig. 3C). Upon brefeldin A (BFA) treatment triggering the collapse of the *A. nidulans* Golgi network into large aggregates [38], GeaA-GFP structures collapse into large aggregates (Fig. 3E). We constructed strains simultaneously expressing GeaA-GFP and the early Golgi marker mCherry-SedV^Sed5^ or the late Golgi marker mRFP-PH^OSBP^ [31], [38], [39] for co-localization analyses (Section 2 and Fig. 4 legend). These showed that GeaA-GFP substantially co-localizes with mCherry-SedV^Sed5^ at the early Golgi, while it largely segregates from mRFP-PH^OSBP^ at the late Golgi (Fig. 4A, C and D). Moreover, GeaA-GFP largely colocalizes with mCherry-SedV in BFA bodies (not shown). These data are consistent with localization at the early-/cis-Golgi reported for members of the GBF/Gea subfamily of Arf1 GEFs (see for example [48]).

We next tagged the *geaA*1 mutant allele with *gfp* (Fig. 3A). GeaA1-GFP is functional as shown by typical *geaA*1 growth of the GeaA1-GFP-expressing strain (Fig. 3B). Moreover, *geaA*1::*gfp* suppresses *hypB*5 thermosensitivity similarly to *geaA*1 (Fig. 3B), establishing that GFP labeling has no detectable effect on GeaA1 function. Like GeaA-GFP, GeaA1-GFP localizes to Golgi-resembling cytosolic structures and the cytosol (Fig. 3C). Strikingly, it also labels, strongly, an apical crescent and an apex-associated material, resembling the localization of the secretory v-snare SynA [49] at the apical plasma membrane crescent, where exocytosis predominates, and at the Spitzenkörper, where post-Golgi carriers accumulate [41]. Plots of fluorescence intensity across the hyphae confirm this GeaA1-GFP redistribution towards the apex (Fig. 3D). Indeed, staining the plasma membrane of GeaA1-GFP-expressing hyphae with the lipophilic dye FM4-64 [50], showed that GeaA1-GFP at the apical crescent is at or very closely associated with the plasma membrane (Fig. 3F). Upon BFA treatment the majority of GeaA1-GFP collapsed in Golgi-like aggregates, but some plasma membrane labeling persisted (Fig. 3E). Using strains co-expressing Golgi markers, we determined that GeaA1-GFP partially co-localizes with the early Golgi marker SedV, although to a lesser extent than GeaA-GFP (Fig. 4B and D). Decrease in co-localization with the early Golgi marker was accompanied by a minor increase in the co-localization with the late Golgi marker (Fig. 4B and D). GeaA1 extensively co-localizes with SedV in BFA bodies (not shown). However, an increase in the proportion of BFA mRFP-PH^OSBP^ late Golgi aggregates containing detectable levels of GeaA1-GFP (87% in *geaA*1 versus 44% in *geaA*) was observed, suggesting some change in Golgi dynamics.

To assess the localization of GeaA1-GFP in a *hypB*5 background, we co-cultivated a *geaA*1::*gfp hypB*5 double mutant and a *hypB*5 single mutant. Double mutants are easily identifiable because of their green fluorescence, but also because at 28 °C *geaA*1::*gfp hypB*5 hyphae are thicker than *hypB*5 hyphae. After a temperature shift to 37 °C, growth of *hypB*5 cells ceased as previously described [31], whereas *geaA*1::*gfp hypB*5 strains continued to grow, confirming that *geaA*1::*gfp* suppresses *hypB*5 in these conditions. Importantly, GeaA1::GFP apical localization is maintained in the double mutant background at 37 °C (Fig. 3C and D), consistent with the possibility that this localization is involved in suppression.

It is largely unknown why two Arf1-GEF subfamilies, asymmetrically located in the Golgi, are conserved among eukaryotes. Our finding that an Arf1-GEF mutation bypasses this asymmetry, rendering a single Arf1-GEF capable of maintaining growth, is unprecedented. GeaA1 partially bypasses HypB. Moreover, *geaA*1 is hypomorphic (or GeaA1 is somewhat deleterious), as *geaA*1 single mutants grow less than wild type. This implies that acquisition of HypB-bypassing capability by GeaA1 occurs at the expense of its physiological role. We have demonstrated that Tyr1022Cys induces a shift in the localization of GeaA1 at the expense of its early Golgi localization. This change in localization appears to be a forward displacement within the secretory pathway, as GeaA1 is, in part, redistributed towards the apical plasma membrane, where exocytic carriers are preferentially delivered. This localization shift might reflect the mutationally altered Golgi dynamics and/or Golgi exit, resulting in re-routing a portion of GeaA1 towards the plasma membrane. Alternatively, the GeaA1 localization shift might reflect a direct role of the Ser-Ω-Φ motif on GeaA localization, in which case the change in localization *per se* would effect the suppression, perhaps by GeaA1 acquiring function at its new apical locale. In this case, one highly speculative possibility is that GeaA1 reorganizes the exocytic pathway in such a way that the Golgi is partially bypassed. Reorganization of the secretory pathway by manipulation of the early Golgi Arf1-GEF has precedents in intracellular membrane remodeling via GBF1 function alteration by RNA viruses [51]. However, despite its minor localization at the late Golgi, we cannot discard the possibility that GeaA1 would have gained function at this compartment. According to this hypothetical possibility, GeaA1 would be capable of performing HypB essential functions at the late Golgi and Arf-GEFs would be, to some extent, interchangeable.

At present we can only speculate on the mechanism by which Tyr1022Cys shifts GeaA localization. The substitution might impair a Ser-Ω-Φ motif-mediated interaction of GeaA with a ‘recycling’ factor/adaptor hypothetically required to restore GeaA localization to the early Golgi during cisternal maturation. This implies that if GeaA1 is inefficiently recycled retrogradely, it must be rapidly sorted into exocytic carriers, as we have only detected a small increase in GeaA1 residence at the late Golgi. A slightly modified alternative is that the motif might bind one key component of a coincidence detection module restricting GeaA localization to the early Golgi. Loss of interaction with this component could make GeaA1 localization largely dependent on a second component with broader distribution. It is notable that a GBF1 HDS1 and HDS2 module containing the Ser-Ω-Φ motif has been shown to redirect GBF1 to the leading cell edge through phosphoinositide binding [52]. Similarly, the GeaA1 mutation might modify a lipid-recognizing module, such that loss of affinity for a Golgi-specific lipid would be accompanied by gain of affinity for an apical crescent-specific lipid, resulting in the shift in GeaA1 localization. In any case, future studies addressing how the GeaA1 substitution has such an impact on its localization constitute a promising tool to understand the mechanisms by which GeaA specifically localizes to the Golgi.

## Appendix A Supplementary data

Supplementary data

Supplementary Table 1 Strains used in this work.

Supplementary Table 2 DNA oligonucleotides used in this work.
