# Supplementary material for: GBF/Gea mutant with a single substitution sustains fungal growth in the absence of BIG/Sec7
Source: FEBS Lett. 2014 Dec 20;588(24):4799–806. doi: 10.1016/j.febslet.2014.11.014 (PMC4266534; doi:10.1016/j.febslet.2014.11.014)
Supplement: Supplementary data [file mmc1.docx]

**Supplementary Materials and Methods**

**Aspergillus nidulans *manipulations***

Strains (Supplementary Table I) carried markers in standard use [[1](#_ENREF_1)]. *Aspergillus* standard complete or minimal medium (CM or MM) [[2](#_ENREF_2)] was used for growth tests and for the selection of suppressors. MM supplemented with biotin, calcium (+)-pantothenate, 1% glucose and 5 mM ammonium tartrate was used for selecting *hypB*5 suppressors. Transformations were carried out as described [[3](#_ENREF_3)].

***Deletion of* geaA**

The cassette for the deletion of *geaA* was obtained from the Fungal Genetics Stock Center (Library clone, ANID_00112.1, 20091001A, C1) [www.fgsc.net, [[4](#_ENREF_4)]] and used to transform MAD1739. For southern blot analysis of heterokaryons, we used as a probe a *geaA* 3´prime sequence amplified with primers 30 and 31.

***Mapping of*** **suA*1*hypB*5 on chromosome VIII; Molecular basis of the* sE*15 mutational lesion***

We mapped *suA*1*hypB*5 on chromosome VIII by meiotic crosses using standard genetic markers. We obtained 2 cM linkage of *suA*1*hypB*5 to *nudA*1 (allele of AN0118) [[5](#_ENREF_5), [6](#_ENREF_6)] and sought genomic markers to determine the orientation of *suA*1*hypB*5 relative to *nudA*1. *nirA*^c/d^101 (allele of AN0098) [[7](#_ENREF_7), [8](#_ENREF_8)], the constitutive, de-repressed allele of *nirA* controlling the nitrate assimilation pathway, is centromere-distal to *nudA*1 and results in hypersensitivity of ClO_3_^-^ toxicity even in the presence of NH4^+^. The *sE*15 sulphate-utilization mutation preventing the reduction of 3'-phosphoadenosine-5'-phosphosulphate (PAPS) to sulphite [[9](#_ENREF_9)] is centromere-proximal to *nirA* on the classical genetic map [[10](#_ENREF_10)], but its genome position had not been determined. Thioredoxin is required for PAPS reductase [see for example [[11](#_ENREF_11)]]. The *A. nidulans* gene encoding thioredoxin A, *trxA* (AN0170, centromere-proximal to *nudA*), has been identified [[12](#_ENREF_12)]. Deletion of *trxA* also leads to requirement for reduced sulphur source [[12](#_ENREF_12)] (M. Thon, P. Hortschansky and A. Brakhage, personal communication). We considered that *sE*15 might be a mutation in *trxA*. Indeed, sequencing of *trxA* in an *sE*15 strain (primers 28 and 29) revealed a nucleotide insertion in *trxA* (c. 382insA: insertion of A after T382 in the cDNA sequence) resulting in addition of 13 out-of-frame residues after wild type Lys127 and truncation of the 189-residue protein. As reduced sulphur sources fully supplement *sE*15 strains, we conclude that the only essential role of thioredoxin in *A. nidulans* is as an intermediate electron carrier in the PAPS reductase reaction. Crossing a strain carrying *sE*15/*trxA*^c.382insA^, *nudA*1 and *nirA*^c/d101^ with a *suA*1*hypB*5 strain, we determined that the suppressor lies between *nudA* and *nirA*.

**References**

1. Clutterbuck, A.J., *Aspergillus nidulans.* Genetic Maps,Vol. 3: Locus Maps of Complex Genomes. Ed. 6, edited by S. J. O'Brien. , 1993. : p. pp. 3.71–3.84, Cold Spring Harbor Laboratory Press, Cold Spring Harbor, NY.

2. Cove, D.J., *The induction and repression of nitrate reductase in the fungus Aspergillus nidulans.* Biochim Biophys Acta, 1966. **113**(1): p. 51-6.

3. Tilburn, J., et al., *Transformation by integration in Aspergillus nidulans.* Gene, 1983. **26**(2-3): p. 205-21.

4. McCluskey, K., *The Fungal Genetics Stock Center: from molds to molecules.* Adv Appl Microbiol, 2003. **52**: p. 245-62.

5. Morris, N.R., *Mitotic mutants of Aspergillus nidulans.* Genet Res, 1975. **26**(3): p. 237-54.

6. Xiang, X., S.M. Beckwith, and N.R. Morris, *Cytoplasmic dynein is involved in nuclear migration in Aspergillus nidulans.* Proc Natl Acad Sci U S A, 1994. **91**(6): p. 2100-4.

7. Burger, G., J. Tilburn, and C. Scazzocchio, *Molecular cloning and functional characterization of the pathway-specific regulatory gene nirA, which controls nitrate assimilation in Aspergillus nidulans.* Mol Cell Biol, 1991. **11**(2): p. 795-802.

8. Rand, K.N. and H.N. Arst, Jr., *Mutations in nirA gene of Aspergillus nidulans and nitrogen metabolism.* Nature, 1978. **272**(5655): p. 732-4.

9. Gravel, R.A. and E. Kafer, *Genetic and accumulation studies in sulfite-requiring mutants of Aspergillus nidulans.* Can J Genet Cytol, 1970. **12**(4): p. 831-40.

10. Clutterbuck, A.J., *The validity of the Aspergillus nidulans linkage map.* Fungal Genet Biol, 1997. **21**(3): p. 267-77.

11. Schwenn, J.D., F.A. Krone, and K. Husmann, *Yeast PAPS reductase: properties and requirements of the purified enzyme.* Arch Microbiol, 1988. **150**(4): p. 313-9.

12. Thon, M., et al., *The thioredoxin system of the filamentous fungus Aspergillus nidulans: impact on development and oxidative stress response.* J Biol Chem, 2007. **282**(37): p. 27259-69.

**Supplementary Figure 1 legend**

**Deletion of the *geaA* gene encoding the only *A. nidulans* early-Golgi Arf1-GEF is lethal.** MAD1739 recipient strain was transformed with a linear DNA fragment containing the *A. fumigatus* *pyrG* flanked by the *geaA* 5´ and 3´UTRs, resulting in *in locus* integration and replacement of the *geaA* coding region by *pyrG*. Primary transformants, selected as pyrimidine prototrophs, were heterokaryotic, carrying both transformed (*geaA*Δ:: *pyrG^Af^*) and wild type (*geaA*+ *pyrG89*) nuclei. The presence of both nuclei in the primary transformants was confirmed by Southern blot, showing both a band of the size corresponding to the wild type *geaA*, as well as the higher mobility band corresponding to *geaA*Δ::*pyrG^Af^*. Spores produced by heterokaryons are uninucleate. On medium without pyrimidines no growth of the prototroph *geaA*Δ::*pyrG^Af^* spores was detected, indicating that GeaA is essential.
