# Supplementary material for: GBF/Gea mutant with a single substitution sustains fungal growth in the absence of BIG/Sec7
Source: FEBS Lett. 2014 Dec 20;588(24):4799–806. doi: 10.1016/j.febslet.2014.11.014 (PMC4266534; doi:10.1016/j.febslet.2014.11.014)
Supplement: Supplementary Table 1 — Strains used in this work. [file mmc2.docx]

| **Supplementary Table I: Strains used in this work** | | |
| --- | --- | --- |
| **MAD collection number** | **Genotype** | **Origin** |
| 2 | *biA*1 | MAD collection |
| 1739 | *pyrG*89; *nkuA*Δ::*bar* *pyroA*4 | Arst HN |
| 2013 | *wA*4*; inoB2 nkuAΔ::bar pyroA*4[*pyroA*-gpdA^mini^::mrfp::PH^OSBP^*]*; niiA*4 | [[1](#_ENREF_1)] |
| 2523 | *biA1 hypB5 pabaA6; chaA1* | *FGSC A1130 [*[*2*](#_ENREF_2)*]* |
| 2808 | *wA*4*; inoB*2 *nkuAΔ::bar pyroA*4[*pyroA*-gpdAmini::mcherry::sed5*]*; niiA*4 | *[*[*3*](#_ENREF_3)*]* |
| 3574 | *hypB*5; *pantoB*100 | *This work* |
| 4041 | *hypB*5; *pantoB*100; *suA*1*hypB*5 | *This work* |
| 4062 | *pabaA*1; *suA*1*hypB*5 | *This work* |
| 4836 | *hypB*5; *pantoB*100; *geaA*1 | *This work* |
| 5107 | *pabaA*1 *pyrG*89; *nkuA*Δ::*bar*; *geaA*1 | *This work* |
| 5130 | *hypB*Δ::*pyrG^Afum^* *pabaA*1 *pyrG*89; *nkuA*Δ::*bar*; *geaA*1 | *This work* |
| 5195 | *pyrG*89; *pyroA*4 *nkuA*Δ::*bar*; *geaA::gfp::3utr::pyrG^Af^* | *This work* |
| 5199 | *pyrG*89 *pabaA*1; *nkuA*Δ::*bar*; *geaA*1::*gfp::3utr::pyrG^Af^* | *This work* |
| 5253 | *pyrG*89*? hypB*5; *nkuA*Δ*::bar?*; *geaA*1*::gfp::3utr::pyrG^Af^* | *This work* |
| 5254 | *pyrG*89?; *inoB*2 *pyroA*4 [*pyroA*::gpdA*^mini^::*mCherry::sed5*] *nkuA*Δ::*bar*; *geaA::gfp::3utr::pyrG^Af^* | *This work* |
| 5256 | *pyrG*89?; *pyroA*4 [*pyroA* gpdA^mini^ mCherry::sed5*] *nkuA*Δ::*bar*; *geaA::gfp::3utr::pyrG^Af^* *niiA*4 | *This work* |
| 5257 | *pyrG*89?; *inoB*2 *pyroA*4[*pyroA*::gpdA^mini^*::*mrfp-PH^OSBP^*] *nkuA*Δ::*bar*; *geaA::gfp::3utr::pyrG^Af^* | *This work* |
| 5258 | *pyrG*89?; *pyroA*4[*pyroA*: gpdA^mini^::mrfp-PH^OSBP^*] *nkuA*Δ::*bar*; *geaA::gfp::3utr::pyrG^Af^* | *This work* |
| 5259 | *pyrG*89?; *pyroA*4[*pyroA* gpdA^mini^ mCherry::sed5*] *nkuA*Δ::*bar*; *geaA*1*::gfp::3utr::pyrG^Af^* | *This work* |
| 5260 | *pyrG*89? *pabaA*1; *pyroA*4 [*pyroA* gpdA^mini^::mCherry::sed5*] *nkuA*Δ::*bar*; *geaA*1::*gfp::3utr::pyrG^Af^ niiA*4 | *This work* |
| 5261 | *pyrG*89?; *pyroA*4[*pyroA*:: gpdA^mini^::mrfp-PH^OSBP^*] *nkuA*Δ::*bar*; *geaA*1*::gfp::3utr::pyrG^Af^ niiA*4 | *This work* |
| 5262 | *pyrG*89?; *pyroA*4[*pyroA*:: gpdA^mini^*::*mrfp-PH^OSBP^*] *nkuA*Δ*::bar*; *geaA*1*::gfp::3utr::pyrG^Af^ niiA*4 | *This work* |

1. Pantazopoulou, A. and M.A. Penalva, *Organization and dynamics of the Aspergillus nidulans Golgi during apical extension and mitosis.* Mol Biol Cell, 2009. **20**(20): p. 4335-47.

2. Yang, Y., et al., *Aspergillus nidulans hypB encodes a Sec7-domain protein important for hyphal morphogenesis.* Fungal Genet Biol, 2008. **45**(5): p. 749-59.

3. Pantazopoulou, A. and M.A. Penalva, *Characterization of Aspergillus nidulans RabC/Rab6.* Traffic, 2011. **12**(4): p. 386-406.
