# Supplementary material for: GBF/Gea mutant with a single substitution sustains fungal growth in the absence of BIG/Sec7
Source: FEBS Lett. 2014 Dec 20;588(24):4799–806. doi: 10.1016/j.febslet.2014.11.014 (PMC4266534; doi:10.1016/j.febslet.2014.11.014)
Supplement: Supplementary Table 2 — DNA oligonucleotides used in this work. [file mmc3.docx]

| **Supplementary Table II: DNA oligonucleotides used in this work** | | |
| --- | --- | --- |
| **Primer identification number** | **Primer name** | **5´to 3´sequence** |
| 1 | *hypB5* Fw | GATCGCGCTCACAAACGTG |
| 2 | *hypB5* Rev | CAGGCAAGTCCTGATTGTC |
| 3 | GSP *sec7* (2) | GCGCCGGCCGACGCTATGCTTGCGAGATGG |
| 4 | GSP *sec7* (1) | CAGCCGAGCACGAGGACGGCGATATGCCAG |
| 5 | MHG_54 Fw1 hypB5 | CATACTCCGGACTATCGCAAGG |
| 6 | MHG_55 Rev1 hypB5 | CCTGTTGCTGCTCGTGGCTG |
| 7 | MHG_56 Fw2 hypB5 | GTAACGAAGACGATGTGGACGAG |
| 8 | MHG_57 Rev3 hypB5 | TGGCCAGAAGACTGAATCTCTTG |
| 9 | MHG_58 Fw3 hypB5 | GGTGAAGGGTGTAGACCGGATC |
| 10 | MHG_59 Fw4 hypB5 | TCATGACTGGCGATGATCTTGAG |
| 11 | MHG_60 Rev4 hypB5 | TGCCTCCAGAGGACATCCCA |
| 12 | MHG_61 Fw5 hypB5 | TCCAGCCTCTGCGTCGACATC |
| 13 | MHG_62 Rev5 hypB5 | CAGCAAACCTCTCACGGAGAGG |
| 14 | GSP 5UTR Sec7 Fw | GGAAAGGAACACCTACAGAGCC |
| 15 | GSP 5UTR Sec7 Rev | GCAAGACTCCGTCTGGAGTATC |
| 16 | Fus 5UTR Sec7 pyrG | CCGATACTCCAGACGGAGTCTTGCACCGGTCGCCTCAAACAATGCTCTTC |
| 17 | GFP Sec7 (B) | CTAAAGCGGATATAAGAGAAGAAAACCAGTCGTCTGAGAGGAGGCACTGATGCG |
| 18 | GSP Sec7 (3) | GACTGGTTTTCTTCTCTTATATCCGCTTTAG |
| 19 | GSP Sec7 (4) | GGCCAAGTCACACATATTGACCGGAACTTC |
| 20 | MHG_8 GeaA-Rev4 | GATCCGCGCTATAAATGCCC |
| 21 | MHG_9 GeaA-Fw4 | GCCCCTTGACTCGCTCAACG |
| 22 | MHG_10 GeaA-Rev3 | CCTTGACAATAGGCACCGCG |
| 23 | MHG_11 GeaA-Fw3 | GCCCAGCCTTCTGAGGTTGC |
| 24 | MHG_12 GeaA-Rev2 | CGGGGGCAAAGTCCTGGC |
| 25 | MHG_13 GeaA-Fw2 | GTCTCTCGGAAGTGCTGCGG |
| 26 | MHG_14 GeaA-Rev1 | GCGACTGTGGTTCCGTCCAC |
| 27 | MHG_15 GeaA-Fw1 | CTGAAGCAAGGAGCTGCACG |
| 28 | Fw TrxA | CTTCTCACGGTTTCGTGCACC |
| 29 | Rev TrxA | CACAAACAAGATACGTCAGGGATG |
| 30 | AP2014-11 FW 3UTR GeaA | CACAATTCATGCCCAGAGAACCTAC |
| 31 | AP2014-15 Rev3prime GeaA | GCTGCCTTGGAACGACAAGCC |
| 32 | AP2014-7 FW ORF GeaA | GCTTGGTCAGCATATTGGTCGC |
| 33 | AP2014-8 Rev ORF GeaA | CTCCCTTTCAGGAGTATCTTGACC |
| 34 | AP2014-9 FWGeaAGFPfus | GAAGGTCAAGATACTCCTGAAAGGGAGGGAGCTGGTGCAGGCGCTGGAGCC |
| 35 | AP2014-10 Rev GeaGFPfus | GTGTAAAGCACATTTGCCAAAATTATCATTATTTGTATAGTTCATCCATGCCATGTG |
| 36 | AP2014-12 Rev3UTRGea | GTTGTTTGGTAATAGTGAGGTGTAGATGTC |
| 37 | AP2014-14 FW3primeGea | CACAATTCATGCCCAGAGAACCTAC |
| 38 | AP2014-16 FWGeapyrGfus | GACATCTACACCTCACTATTACCAAACAACACCGGTCGCCTCAAACAATGCTCTTC |
| 39 | AP2014-17 RevGeapyrGfus | GTGTAGGTTCTCTGGGCATGAATTGTGCTGTCTGAGAGGAGGCACTGATGCG |
